# Supplementary material for: Protein destabilization underlies pathogenic missense mutations in ARID1B
Source: Nat Struct Mol Biol. 2024 Feb 12;31(7):1018–22. doi: 10.1038/s41594-024-01229-2 (PMC11257965; doi:10.1038/s41594-024-01229-2)
Supplement: Supplementary file 1 — Reporting Summary [file 41594_2024_1229_MOESM1_ESM.pdf]

nature portfolio

Corresponding author(s): Giorgio Galli and Fabian Sesterhenn

Last updated by author(s): Dec 22, 2023

Reporting Summary

Nature Portfolio wishes to improve the reproducibility of the work that we publish. This form provides structure for consistency and transparency in reporting. For further information on Nature Portfolio policies, see our Editorial Policies and the Editorial Policy Checklist.

Statistics

For all statistical analyses, confirm that the following items are present in the figure legend, table legend, main text, or Methods section.

n/a	Confirmed	
☐	☒	The exact sample size (*n*) for each experimental group/condition, given as a discrete number and unit of measurement
☐	☒	A statement on whether measurements were taken from distinct samples or whether the same sample was measured repeatedly
☐	☒	The statistical test(s) used AND whether they are one- or two-sided<br>*Only common tests should be described solely by name; describe more complex techniques in the Methods section.*
☒	☐	A description of all covariates tested
☐	☒	A description of any assumptions or corrections, such as tests of normality and adjustment for multiple comparisons
☐	☒	A full description of the statistical parameters including central tendency (e.g. means) or other basic estimates (e.g. regression coefficient) AND variation (e.g. standard deviation) or associated estimates of uncertainty (e.g. confidence intervals)
☐	☒	For null hypothesis testing, the test statistic (e.g. *F*, *t*, *r*) with confidence intervals, effect sizes, degrees of freedom and *P* value noted<br>*Give P values as exact values whenever suitable.*
☒	☐	For Bayesian analysis, information on the choice of priors and Markov chain Monte Carlo settings
☒	☐	For hierarchical and complex designs, identification of the appropriate level for tests and full reporting of outcomes
☒	☐	Estimates of effect sizes (e.g. Cohen's *d*, Pearson's *r*), indicating how they were calculated

Our web collection on statistics for biologists contains articles on many of the points above.

Software and code

Policy information about availability of computer code

Data collection	Standard software from manufacturers (Illumina Inc., Illumina NovaSeq control software Version 1.8.1) for NGS-based data collection has been used. Additionally basecalling was performing with RTA v3.4.4. Post-run processing was performed with BCL2FASTQ v2.20.0.422. Run QC was performed with FASTQC v0.11.9.<br>For Flow Cytometry based assay: CytExpert 2.4.0.28 for analysis and BD FACSDiva™ Software v9.0 for FACS sorting
Data analysis	Statistics were calculated by Bioconductor (v. 3.16) R packages.<br>For NGS data analysis, open source code have been used and listed in the material and methods. Additionally, the entire code for pipeline and data analysis including version control has been deposited in Github https://github.com/Novartis/dms-pipeline/tree/main and Zenodo under record 10418664.

For manuscripts utilizing custom algorithms or software that are central to the research but not yet described in published literature, software must be made available to editors and reviewers. We strongly encourage code deposition in a community repository (e.g. GitHub). See the Nature Portfolio guidelines for submitting code & software for further information.

Data

Policy information about availability of data

All manuscripts must include a data availability statement. This statement should provide the following information, where applicable:

- Accession codes, unique identifiers, or web links for publicly available datasets
- A description of any restrictions on data availability
- For clinical datasets or third party data, please ensure that the statement adheres to our policy

All the data have been to SRA with BioProject ID: PRJNA1010676 and can be publicly accessed here ID 1010676 - BioProject - NCBI (nih.gov). All the processed data can additionally be found as supplementary tables and deposited in Zenodo under record 10418664.

Research involving human participants, their data, or biological material

Policy information about studies with human participants or human data. See also policy information about sex, gender (identity/presentation), and sexual orientation and race, ethnicity and racism.

Reporting on sex and gender	not applicable
Reporting on race, ethnicity, or other socially relevant groupings	not applicable
Population characteristics	not applicable
Recruitment	not applicable
Ethics oversight	not applicable

Note that full information on the approval of the study protocol must also be provided in the manuscript.

Field-specific reporting

Please select the one below that is the best fit for your research. If you are not sure, read the appropriate sections before making your selection.

☒ Life sciences ☐ Behavioural & social sciences ☐ Ecological, evolutionary & environmental sciences

For a reference copy of the document with all sections, see nature.com/documents/nr-reporting-summary-flat.pdf

Life sciences study design

All studies must disclose on these points even when the disclosure is negative.

Sample size	Experiments were performed using sample sizes based on standard protocols in the field (n >= 3 for standard cell biology experiments to enable two sided t-tests for normally distributed data). No statistical test was performed to predetermine sample size.
Data exclusions	No data were excluded.
Replication	All experiments were repeated at least for three times. Detailed information on replicates was available in the figure legends. All attempts to replicate the experiments performed here were successful.
Randomization	Samples were processed in a randomized fashion.
Blinding	Data acquisition in the studies was conducted in a blinded manner. Data processing was blinded by assigning a random ID constituted of 3 letters and 3 numbers. For analyzing contrasts (e.g. NGS data from different sorted populations or Treated vs. Untreated) the data analyst was informed about the nature of the sample to enable the analysis.

Reporting for specific materials, systems and methods

We require information from authors about some types of materials, experimental systems and methods used in many studies. Here, indicate whether each material, system or method listed is relevant to your study. If you are not sure if a list item applies to your research, read the appropriate section before selecting a response.

Materials & experimental systems

n/a	Involved in the study
☐	☒ Antibodies
☐	☒ Eukaryotic cell lines
☒	☐ Palaeontology and archaeology
☒	☐ Animals and other organisms
☒	☐ Clinical data
☒	☐ Dual use research of concern
☒	☐ Plants

Methods

n/a	Involved in the study
☒	☐ ChIP-seq
☐	☒ Flow cytometry
☒	☐ MRI-based neuroimaging

Antibodies

Antibodies used

Antibodies used:
Actin (Millipore, MAB1501; 1:1000 dilution), HA (Cell Signaling, 3724; 1:1000 dilution), ARID1B (Sigma, WH0057492M1, 1:500 dilution), SMARCB1 (Cell signaling, 91735, 1:1000 dilution), BRG1 (Abcam, ab110641, 1:1000 dilution) and HRP-anti-rabbit and HRP-anti-mouse (Cell Signaling, 1:2500 dilution).

Validation

Antibodies were validated by RNAi/CRISPR experiments (western blot upon siRNA or shRNA knockdown or CRISPR KO, data not shown) or cell lines diplaying differential expression.
Actin: https://www.merckmillipore.com/CH/de/product/Anti-Actin-Antibody-clone-C4,MM_NF-MAB1501
HA: https://www.cellsignal.com/products/primary-antibodies/ha-tag-c29f4-rabbit-mab/3724
ARID1B: https://www.sigmaaldrich.com/CH/de/product/sigma/wh0057492m1 and RNAi in Fig S2A
SMARCB1: https://www.cellsignal.com/products/primary-antibodies/smarcb1-baf47-d8m1x-rabbit-mab/91735
BRG1 (SMARCA4):
https://www.abcam.com/products/primary-antibodies/brg1-antibody-epncir111a-ab110641.html

Eukaryotic cell lines

Policy information about cell lines and Sex and Gender in Research

Cell line source(s)

HEK-293a cells were obtained from Thermo Fisher (R70507) and Cal-51 were obtained from DMSZ (ACC 302).
HEK293 ARID1A/B dKO cells were generated by transfecting all-in-one CRISPR plasmids expressing the following sgRNA sequences: sgARID1B_2 (5′-ACCGTGAGGTGCCAACGTTTAGGT-3′) sgARID1B_3 (5′-ACCGAAACTTGATAAGCTTCCTAG-3′), sgARID1B_8 (5′-ACCGGGCACCCCACTATACGCTGG-3′), sgARID1A_2 (5′-ACCGTTGAGATGTCCAAACACCCA-3′), sgARID1A_3 (5′-ACCGGATGTTGGCGAGTGTAACCA-3′), sgARID1A_4 (5′-ACCGCTTGCAACCAACCTCAATGT -3′).

Authentication

Cell line identity was confirmed by regular SNP array genotyping

Mycoplasma contamination

Cell lines were regularly tested for mycoplasma contamination and cell lines were confirmed to be negative before using for experiments

Commonly misidentified lines
(See ICLAC register)

No commonly misidentified cell lines were used

Flow Cytometry

Plots

Confirm that:

☒ The axis labels state the marker and fluorochrome used (e.g. CD4-FITC).

☒ The axis scales are clearly visible. Include numbers along axes only for bottom left plot of group (a 'group' is an analysis of identical markers).

☒ All plots are contour plots with outliers or pseudocolor plots.

☒ A numerical value for number of cells or percentage (with statistics) is provided.

Methodology

Sample preparation

Cells were trypsinized and resuspended

Instrument

BD Cytoflex LS for analysis and BD FACSAria™ Fusion for FACS sorting

Software

CytExpert 2.4.0.28 for analysis and BD FACSDiva™ Software v9.0 for FACS sorting

Cell population abundance

Within the "single cells" population, we report % of cells falling into the gate of cells displaying lower GFP levels than mCherry (deviating from the diagonal).

Gating strategy

Live cells were first gated based on FSC and SSC to exclude debris. After single cells were gated based on FSC-height and FSC-width. Out of the single cells population the cells deviating from the diagonal in the scatter plot comparing mCherry (in the FL11 channel) and GFP (in the FL1 channel) were quantified. See exemplary figure in Extended Data S2B.

☒ Tick this box to confirm that a figure exemplifying the gating strategy is provided in the Supplementary Information.
