# Supplementary figures and images for: Protein destabilization underlies pathogenic missense mutations in ARID1B
Source: Nat Struct Mol Biol. 2024 Feb 12;31(7):1018–22. doi: 10.1038/s41594-024-01229-2 (PMC11257965; doi:10.1038/s41594-024-01229-2)

Uncropped blots

Fig. 1F

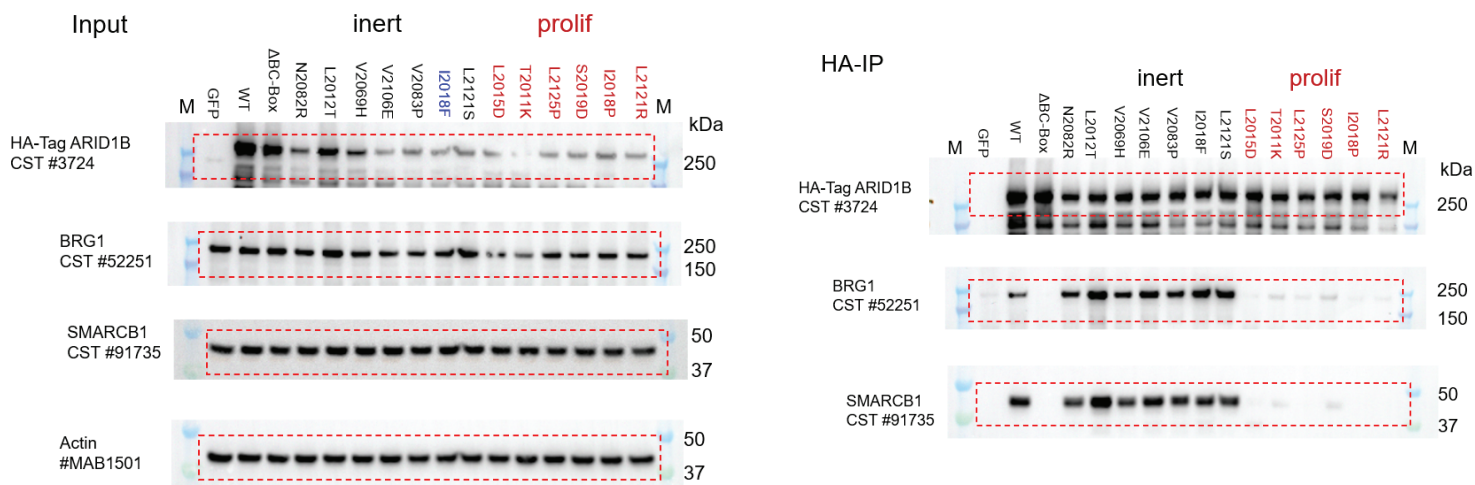

Extended data fig. 2A

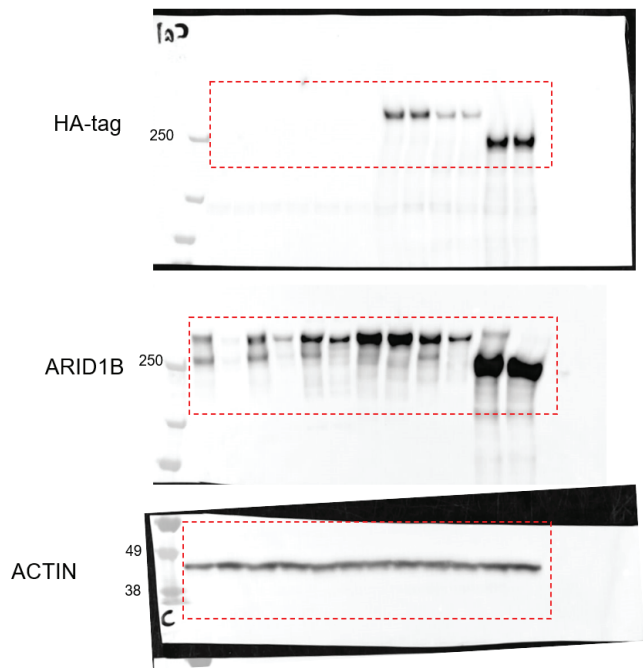

Extended data fig. 8B

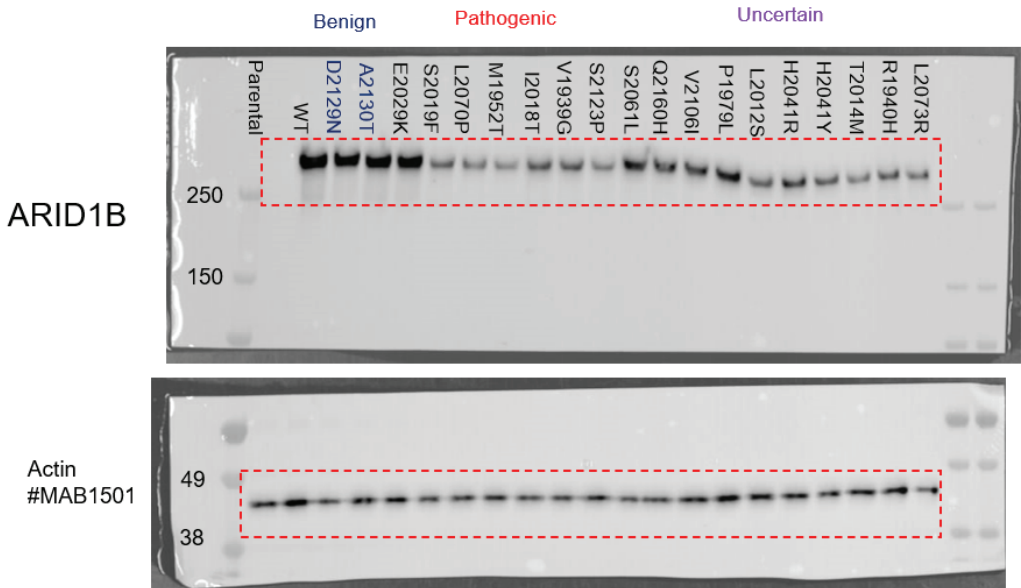

Extended data fig. 8E

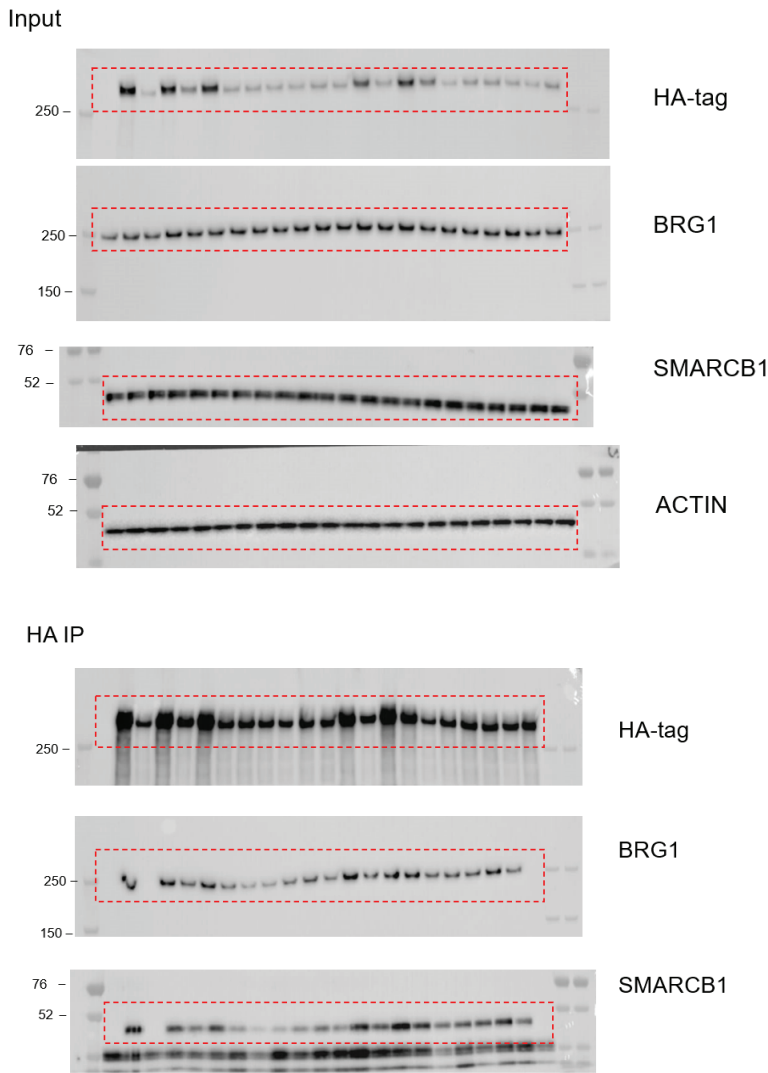

Supplement: Supplementary file 4 — Uncropped blots. [file 41594_2024_1229_MOESM4_ESM.pdf]
